# qsubsec: a lightweight template system for defining sun grid engine workflows

**DOI:** 10.1093/bioinformatics/btv698

**Published:** 2015-12-03

**Authors:** Alastair P. Droop

**Affiliations:** MRC Medical Bioinformatics Centre, University of Leeds, Clarendon Way, Leeds LS2 9NL, UK

## Abstract

**Summary:** The Sun Grid Engine (SGE) high-performance computing batch queueing system is commonly used in bioinformatics analysis. Creating re-usable scripts for the SGE is a common challenge. The qsubsec template language and interpreter described here allow researchers to easily create generic template definitions that encapsulate a particular computational job, effectively separating the process logic from the specific run details. At submission time, the generic template is filled in with specific values. This system provides an intermediate level between simple scripting and complete workflow management tools.

**Availability and implementation:** Qsubsec is open-source and is available at https://github.com/alastair-droop/qsubsec.

**Contact:** a.p.droop@leeds.ac.uk

**Supplementary information:** Supplementary data are available at *Bioinformatics* online.

## 1 Introduction

High-performance computing (HPC) is fast becoming an essential part of all but the smallest bioinformatics analyses. Increasingly, shared HPC resources are available for research use. Although a great asset to bioinformatics, these systems are usually shared across multiple disciplines and are utilized by multiple simultaneous users. Such multi-user, multi-application systems require scheduling software to ensure fair use of the resources between multiple users and to make sure that the resources are optimally utilized. These schedulers demand researchers submit their analyses in a specific format with suitable metadata on memory and time limits. The Sun Grid Engine (SGE: https://arc.liv.ac.uk/trac/SGE) is a popular version of such a system.

To make full use of the available HPC, researchers must adapt their analysis scripts to work with the associated scheduling systems. Frequently, this involves breaking down large or time consuming analyses into smaller steps to make better use of resource limitations imposed by the HPC, or to parallelise independent tasks. Ordered collections of individual computational steps constitute a protocol or workflow.

Workflow management software, for example Taverna Workbench (Wolstencroft *et al.*, 2013), Pegasus (Deelman *et al.*, 2015) and Galaxy (Giardine *et al.*, 2005), aims to simplify the creation, management and execution of workflows. These systems attempt to be complete data management and integration tools. As such, they allow users to search online data repositories, download relevant data and provide sets of common analysis tools (Deelman *et al.*, 2009). These systems ultimately attempt to simplify common bioinformatics analyses but there can be several major hurdles: installation and maintenance are not trivial, workflow definitions often require knowledge of languages such as CWL (https://github.com/common-workflow-language/common-workflow-language) or SCUFL (Oinn *et al.*, 2004), and the user is conceptually removed from the running code. Furthermore, the multi-application nature of HPC often precludes installation of complex domain-specific software suites; in these cases, a full workflow management suite might well not be available.

In many cases, these complex management suites are unnecessary, and a simpler framework for defining a workflow element is required. I here describe qsubsec, a Python-based mini-language that separates the core logic of a computational task from the specific data for a single instance without the overhead of a workflow management system. This enables users to easily write SGE job scripts in a generic form that is processed at submission time into a specific computational task. This greatly simplifies the process of defining and maintaining computational pipelines on general-purpose HPC systems.

## 2 Implementation

The qsubsec template mini-language and interpreter allow the computational logic of a job, along with the metadata required by the SGE queueing system, to be defined in a simple, generalised form. Specific data (for example sample names or identifiers) are provided to the interpreter at submission time. The template and specific data are then used to create and submit a job to the SGE queue. This system allows simple scripts written for use in the terminal to be trivially converted to qsubsec template files.

By separating computational logic and specific data, template files can be reused in different analyses saving a large amount of code repetition. Generalization is implemented using tokens; a token (specified as an uppercase identifier surrounded by curly braces) is used wherever a run-specific data value is required. Tokens can be used in all parts of the template, including the scheduler resource definitions and job names. At submission time, the script is parsed and the values of all tokens present in the template file provided by the user.

The qsubsec tool temporarily appends the specific qsubsec language functions (such as section, limits and command) to the Python 3 built-in function list and parses the supplied template using this superset. This allows the template scripts to utilize the full power of Python during job pre-processing. For example, data can easily be read from web servers or from SQL databases before the parsed job is submitted to SGE. A full description of the qsubsec built-in functions is provided in the Supplementary Material.

### 2.1 Example usage

**Fig. 1:**
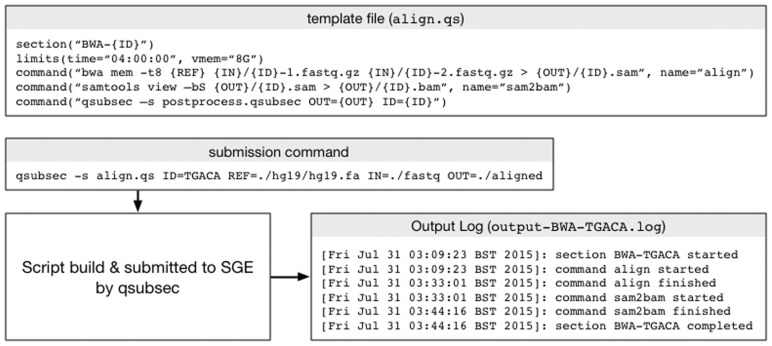
Example template file syntax and submission. A bwa alignment of a pair of samples followed by SAM to BAM conversion is shown. The template file does not contain specific file names. Upon submission, the script is filled in with user-supplied token values. More complex examples are provided in the online project repository

Alignment to a reference genome using bwa (Li and Durbin, 2009) is a common task. Figure 1 shows a simple bwa alignment task (alignment and conversion of the output SAM file to BAM format) implemented as a qsubsec template file. The reference used, the relevant data directories and the sample ID are all expressed as tokens. More complicated examples are provided in the project repository.

The section command defines the job section, and provides a job name. The limits command specifies the limits that SGE will allocate to the job. Each command function defines executable code that will be run. In this example, the third command submits a subsequent template to the queue. By default, script error and output files are captured in files with names based upon the section ID. As the processor is a Python 3 script, there are very few dependencies to install on the HPC system used. This allows extremely simple deployment on mixed use machines, as there is a very low installation and maintenance overhead.

Although developed and tested on SGE, multiple scheduling systems (such as the Open Grid Engine) use a very similar syntax. The scripts generated by qsubsec are likely to be compatible with these other systems.

### 2.2 Iterated tokens

If a series of related jobs are to be submitted with different parameters, multiple values for each token can be provided. The interpreter builds a job script for each unique combination of token values.

### 2.3 Logging

All jobs submitted to SGE have both standard output and error pipes. The qsubsec system catches these and writes them to files named based upon the job ID. By default, qsubsec appends time-stamped progress messages to the output file (corresponding to stdout), allowing job progress to be monitored. Many SGE implementations will provide a short warning time before terminating a job that has overrun its allocated resources. Imminent job terminating is also recorded to the output file. Each command can optionally be tested for successful completion, and the whole job halted on failure. Figure 1 shows example output from the alignment section.

## 3 Summary

Here I describe qsubsec, a mini-language and interpreter to describe grid jobs on the SGE queueing system. The interpreter is very easy to use and requires minimal installation on HPC systems. Existing computational tasks can be easily converted into generic qsubsec templates. The qsubsec system provides a simple system that allows researchers to write generic job templates and thus to run their analyses on high-performance computing systems using SGE. The reporting system ensures analysis workflows, including software versions, are adequately captured.

## Supplementary Material

Supplementary Data
