# Supplementary material for: qsubsec: a lightweight template system for defining sun grid engine workflows
Source: Bioinformatics. 2015 Dec 3;32(8):1267–8. doi: 10.1093/bioinformatics/btv698 (PMC4824124; doi:10.1093/bioinformatics/btv698)
Supplement: Supplementary Data [file supp_32_8_1267__index.html]

qsubsec: A lightweight template system for defining Sun Grid Engine workflows — qsubsec: a lightweight template system for defining sun grid engine workflows — qsubsec: a lightweight template system for defining sun grid engine workflows — Supplementary Data 

# qsubsec: a lightweight template system for defining sun grid engine workflows

## Supplementary Data

files

- Supplementary Data - docx file
