# Supplementary material for: qsubsec: a lightweight template system for defining sun grid engine workflows
Source: Bioinformatics. 2015 Dec 3;32(8):1267–8. doi: 10.1093/bioinformatics/btv698 (PMC4824124; doi:10.1093/bioinformatics/btv698)
Supplement: Supplementary Data [file supp_btv698_qsubsec-supplemental-data.docx]

The qsubsec Template System

# Summary

Most high-performance computing uses a queueing system for allocation of resources to requested jobs. Son of Grid Engine (SGE) is a very popular and prevalent queueing system. The basic SGE usage model is that a user will submit a bash script to the queue using the qsub command. A qsub script contains both the commands to be run as well as details of requested resources, input and output redirects and other options to the queueing system. Frequently in scientific computing, a specific task must be repeated multiple times for different samples or parameter sets. The qsubsec language allows users to create generic qsub scripts that contain placeholders (tokens) that are filled in with specific values at submission time. This allows users to write workflow scripts for the SGE system without the overhead of complex workflow management software.

A qsubsec template file is interpreted as Python script with a few extra builtin commands. This allows template files to contain complex logic that is processed at submission time.

# The qsubsec Template Language

A qsubsec template file defines a single section that is tokenised to generate one or more valid qsub scripts. The qsubsec language is a superset of Python3, so any valid Python code can be included. At submission, the specific values for each token are provided, the section file is run as a Python script, and a valid qsub script file is produced. If multiple values of a token are given, then multiple qsub scripts are generated.

## Tokens

Tokens are very simple text placeholders. In templates, tokens are defined as an uppercase string surrounded by curly braces. Whitespace is not allowed in template names.

## Commands

The qsubsec language is a superset of Python3. The following extra builtins are provided:

section(name, description=None)

Define the section. The name is character field that is used to identify a qsub job (using the -N option). If provided, the description is used to identify the template. Only a single section is possible per section file; only the last processed section command is used.

limits(time, vmem=None, nodes=None)

Define the limits imposed by qsub on the submitted job. The time constraint is in the form ‘hh:mm:ss’, and is mandatory. The vmem constraint is a string defining the memory limit for the job, in the format speed by qsub (e.g. “1G”). The nodes constraint asks qsub for a whole node, and thus overrides the vmem constraint. The nodes parameter is an integer.

option(switch, argument=None, value=None)

Pass further options to qsub. Options are in the form “-switch” (e.g. -V) “-switch argument” (e.g. -m be), or “-switch argument=value” (e.g. -l cputype=amd). Switches should be specified without the initial hyphen. In all cases, the arguments and values are treated as text.

options(options)

Pass a list of options as simple strings.

hold(hold_id)

Add a hold constraint to the job. If provided, the hold_id is treated as a job ID, and the submitted job will wait until there is no job with the specified name in the queue before running.

require(value, flag)

Add requirement checks to a qsub script. Requirements are implemented as blocks of bash code within the qsub script, so are checked once the job is running rather than at submission time. Possible flag strings are are:

path_absent The specified path must be absent

path_present The specified path must be present

path_readable The specified path must be readable

path_writable The specified path must be writable

env_set The specified environment variable must be set

env_unset The specified environment variable must not be set

output(base, file=None)

Set the job output log file. By default, the output log is created in the current directory, and is named “output-N.log” where N is replaced by the name of the job.

error(base, file=None)

Set the job error log file. By default, the error log is created in the current directory, and is named “error-N.log” where N is replaced by the name of the job.

command(cmd, name=None, test=True, log=True)

Adds a command to the script. The command is specified by cmd. If name is specified, the command is named in the output logs, otherwise commands are given a number. If test is True, then script will test for successful completion of the command and generate an error if it failed. If log is True, logging text is produced in the output file.

## Option Types

As options, limits, output & error files and holds all yield qsub options, they can be passed as options. There is difference between the two approaches. For example, extra constraints can be specified as options.

## Example Template Script

The following example template script shows many of the qsubsec features:

section("{NAME}_{STRING}", "This is a test script")
limits(time="00:00:10", vmem="10M")
options(["V", "cwd"])
command("""echo "{STRING}" > ~/{NAME}_{STRING}.txt""", test=True, log=True)

When the template is processed with the {NAME} token given the value “N” and the {STRING} token the value “Hello”, the resulting qsub script is:

#$ -N N_Hello
#$ -l h_rt=00:00:10
#$ -l h_vmem=10M
#$ -V
#$ -cwd
#$ -o ./output-N_Hello.log
#$ -e ./error-N_Hello.log
echo "[`date`]: section N_Hello started"
echo "[`date`]: command 0x1 started"
echo "Hello" > ~/N_Hello.txt || { echo "[`date`]: command 0x1 failed"; exit 1; }
echo "[`date`]: command 0x1 finished"
echo "[`date`]: section N_Hello completed"

# The qsubsec command

Template files are processed using the qsubsec command. The basic form requires a template file and the values of each token specified in the template. Token definitions are given on the command line as TOKEN=value, without the curly braces around the token name. A set of command line arguments can be read from file (one argument per line) by prefixing the file name with an at-sign at the end of the command line. This allows standard token values to be stored in a file and reused by multiple submissions.

## Iterated Tokens

Iterated tokens allow a token to have multiple specified values. Iterated tokens can be specified on the command line as either -iTOKEN=value_1,value_2,…,value_n or -iTOKEN=filename. In the first form, multiple token values are comma-separated. In the second form, the specified file is read with a single token value per line. A qsub script is generated for each unique combination of tokens, so multiple iterated tokens can yield very large numbers of generated scripts. If more than one set of tokens is supplied, qsubsec will not print out the generated script to the console. If automatic submission is not requested, the script will simply enumerate the token sets that would be submitted.

## Automatic Submission

If a single qsub script is generated (i.e. no iterated tokens are specified), the resulting qsub script is printed to the console. However, if qsubsec is invoked from a machine that can submit queue jobs, qsubsec can automatically submit the script, rather than displaying it. In this case, the output from the submission executable is displayed.

## Command Arguments

The arguments that can be passed to qsubsec are:

| -h | —help | show the help information |
| --- | --- | --- |
| -v | —version | show the version number |
| -w | —debug | display extra data for Python errors inside template files |
| -e | —executable | Set the executable used to submit jobs |
| -i | —iterate | Set an iterated token |
| -d | —description | Print the description for a template rather than tokenising it |
| -t | —tokens | Display the tokens referenced in a template |
| -c | —show-code | Process a template file and display its simplified qsubsec code |
| -s | —submit | Submit a processed template |
